# YANA – a software tool for analyzing flux modes, gene-expression and enzyme activities

**DOI:** 10.1186/1471-2105-6-135

**Published:** 2005-06-01

**Authors:** Roland Schwarz, Patrick Musch, Axel von Kamp, Bernd Engels, Heiner Schirmer, Stefan Schuster, Thomas Dandekar

**Affiliations:** 1 Dept of Bioinformatics, Biocenter, University of Würzburg; Germany; 2 Dept of Theoretical Chemistry, Organikum, University of Würzburg, Germany; 3 Dept of Bioinformatics, University of Jena, Germany; 4 Center for Biochemistry (BZH), University of Heidelberg, Germany; 5 Structural and Computational Biology, EMBL, Heidelberg, Germany

## Abstract

**Background:**

A number of algorithms for steady state analysis of metabolic networks have been developed over the years. Of these, Elementary Mode Analysis (EMA) has proven especially useful. Despite its low user-friendliness, METATOOL as a reliable high-performance implementation of the algorithm has been the instrument of choice up to now. As reported here, the analysis of metabolic networks has been improved by an editor and analyzer of metabolic flux modes. Analysis routines for expression levels and the most central, well connected metabolites and their metabolic connections are of particular interest.

**Results:**

YANA features a platform-independent, dedicated toolbox for metabolic networks with a graphical user interface to calculate (integrating METATOOL), edit (including support for the SBML format), visualize, centralize, and compare elementary flux modes. Further, YANA calculates expected flux distributions for a given Elementary Mode (EM) activity pattern and vice versa. Moreover, a dissection algorithm, a centralization algorithm, and an average diameter routine can be used to simplify and analyze complex networks. Proteomics or gene expression data give a rough indication of some individual enzyme activities, whereas the complete flux distribution in the network is often not known. As such data are noisy, YANA features a fast evolutionary algorithm (EA) for the prediction of EM activities with minimum error, including alerts for inconsistent experimental data. We offer the possibility to include further known constraints (e.g. growth constraints) in the EA calculation process. The redox metabolism around glutathione reductase serves as an illustration example. All software and documentation are available for download at .

**Conclusion:**

A graphical toolbox and an editor for METATOOL as well as a series of additional routines for metabolic network analyses constitute a new user-friendly software for such efforts.

## Background

### Elementary mode analysis (EMA) analyzes complex metabolic networks

Metabolic networks include many enzymes. These operate together in a complex way as metabolites of one reaction may be processed (consumed or provided) by a number of different enzymes. Whereas in biochemistry textbooks such networks are often described as linear pathways or simple, separate subnetworks, real metabolic webs show an astonishing complexity regarding the number of possible routes a metabolite can take through the network.

EMA is an algorithm that systematically enumerates all possibilities how enzymes can operate together without violating the steady state condition of the system (see below). Using EMA, complex networks can be analyzed in terms of contained pathways, robustness, central enzymes, medical targets, optimum yield and effector compounds, such as signaling phospholipids, with interesting applications in medicine and biotechnology [1].

### EMA – algorithm and related approaches

To perform a holistic network analysis, the stoichiometric and thermodynamic feasibility of all possible pathways has to be tested. We therefore assume the system to be in a steady-state, in which intermediate or internal metabolites are balanced [2]. Their concentrations do not change in the timescale of study as the amount of production of these metabolites equals their consumption.

To find all pathways through a given network we look for all vectors *v *of enzyme coefficients, the so called flux vectors or flux distributions, which satisfy the steady-state condition of

N*v = 0     (1)

for all internal metabolites (stoichiometric feasibility). Here, *N *is the *m *× *r *stoichiometric matrix of the system with *m *being the number of metabolites in the system and *r *being the number of reactions (in eq. (4), upper case R is used). To solve such systems under consideration of additional irreversibility constraints imposed by the reactions in the system (thermodynamic feasibility), the mathematical theory of convex analysis [3] is used to project the equation above and the irreversibility constraints into what is called a pointed convex polyhedral cone. This approach is used by several algorithms to determine the possible pathways through the system, out of which recent analyses have focused on two concepts [4]: Extreme Pathways [5] and Elementary Mode Analysis (EMA) [2].

Both algorithms return the edges of the calculated cone, the convex basis, as pathways. In addition, EMA returns all possible non-decomposable pathways through the network, the so called Elementary Modes (EMs) or (Elementary) Flux Modes. Both methods yield a complete description of the metabolic network in which every concrete "state" of the system can be described as a non-negative linear combination of the set of pathways or EMs returned.

Elementary Mode Analysis has been successfully applied to numerous biochemical systems [6-8] and its capability to determine maximum conversion yields [9] and minimal cut sets [10] of biochemical systems makes it an important tool to predict the effect of enzyme inactivations, pharmacological effects, growth calculations and biotechnological applications [11]. We previously developed the software METATOOL [12] as an implementation of the Elementary Mode Analysis and enhanced EMA further by developing new techniques to cope with complex networks. These include the dissection of the network at metabolites with especially high connectivity [13] and an approach to reduce the complexity of the network by systematic variation of the internal and external status of the involved metabolites, thus reducing the number of EMs returned [14].

### Development and advantages of YANA

METATOOL represents an efficient implementation of the EMA algorithm and has been integrated as an analysis option in large software packages such as GEPASI [15]. However, as a command line driven program, it lacks the comfort and usability of a graphical user interface (GUI) as well as the ability to perform further analyses out of the box. Alternatively, phpMetatool [16] provides some predefined biochemical networks but offers no further analysis options or processing of the METATOOL results. The program FluxAnalyzer [17] provides a graphical interface and some processing of elementary modes, for example, computation of minimal cut sets [10]. For dissection of complex networks, other tools such as SEPARATOR [13] have to be installed and import and export data using the METATOOL text file format. This gives rise to several common data-exchange and formatting problems.

YANA offers now an integrated modeling environment with standardized data exchange capabilities. It is a platform for integrating future analysis modules and includes strategies to address one of the most important issues in current metabolic modeling, the combinatorial explosion of EMs in complex networks. Further, it allows the convenient modification editing of metabolic networks with a comfortable user interface and the possibility of performing EMA analyses using the established METATOOL algorithm. It additionally incorporates strategies to reduce network complexity by using threshold operations on the metabolites and brings a variety of visualization options for concrete flux distributions of a network. It calculates for a user-defined EM activity pattern the resulting flux distribution, and is further able to predict a valid EM activity pattern from a given flux distribution even when only few or inaccurate enzyme activity data are available from experiments.

## Implementation

### Elementary Mode Analysis

To perform pathway analysis on the network under study, YANA acts as a front-end to METATOOL and computes the Elementary Modes of a network if the following information is provided:

• Metabolites used in the system, including information whether they are treated as internal or external

• Enzymes / reactions involved in the network, including their substrates and products and irreversibility constraints

Parsing the output from METATOOL, YANA shows a tabular overview of the calculated EMs and is able to display detailed information for each of the EMs obtained, including partaking reactions, their reaction equations, as well as the overall net reactions of the Elementary Modes.

Additionally the average diameter (path length) of the EMs is displayed, an information of importance for the dissection of larger networks.

### Reducing network complexity

To prevent combinatorial explosion of the number of EMs in well connected networks, YANA offers and implements two strategies to reduce network complexity. Both change systematically the internal / external status of the metabolites using their connectivity values as the basic criterion.

In the first strategy [13], the network is divided by automatically setting metabolites with a connectivity value above a certain user-defined threshold as "external". This results in a split of the metabolic network into sub-networks, which are convenient to analyze. Individual sub-networks can be obtained using the program SEPERATOR [13], and the new routine from YANA directly gives the resulting complete but simplified network.

Alternatively, YANA offers the option to set all metabolites with a connectivity value below the threshold as external. In this way, only connections between the core nodes of a metabolic system are included, neglecting those on the outskirts. The resulting pathway set still holds the most important EMs, shortened and focused on the central hub metabolites [18].

To get an estimate on the average size of the metabolic network before and after dissection the average diameter (path length) for the modes can be used.

### Translating EM activities into flux distributions

As described in the background section, convex analysis returns the spanning vectors of the cone that describes the solution to the steady-state equation system and thus every actual flux distribution (vector *v *in equation 1) is a linear combination of the obtained EMs.

By assigning an activity value in percent to each EM, except for a scalar factor, every flux distribution possible for the system can be reached. These valid flux distributions, or flux vectors *v*, hold an integer value for each enzyme in the system. Those values, which are responsible for sustaining the steady state in the system (*v *satisfies equation 1 for all internal metabolites) represent the relative flux through the respective enzyme and thus must be a combination of both the reaction velocity of the enzyme (real enzyme kinetics) and the amount of protein available.

YANA offers the possibility to compute flux distributions both in absolute (ignoring reaction directions) and relative values. This is done by iterating over all calculated modes, and summing the absolute (eq. 2) or relative (eq. 3) flux coefficients of each enzyme multiplied with the activity of the EM.





The resulting flux distribution is visualized and presented to the user either in form of graphical bar and pie charts (Figure 2) or in tabular form (Table 4).

Calculating flux distributions from a given theoretical set of EM activities is important e.g. to estimate the relevance of an enzyme in a metabolic network [19], but it would be a desirable goal if one could somehow measure flux distributions in living cells and map them onto the EM activities [20]. It would then be possible to take an experimental snapshot of the system and from it derive actual pathway activities.

### Translating (partially) known flux distributions into EM activities

To obtain flux distributions of a living cell, one could either measure metabolite fluxes directly or estimate fluxes from protein quantification and enzyme turnover rates. Protein amounts will, in practice, be measured either by proteomics or gene expression. For the latter, an estimate from a comprehensive RNA and protein expression analysis in yeast indicates that for each mRNA copy on average there are 4000 molecules of synthesized protein found [21], with individual variation depending on mRNA stability, translatory regulation and promotor activities. To measure all these different factors involved in expression levels and, further, the enzyme activity itself is a non-trivial undertaking. There is a complex interplay between mRNA expression level, protein expression level, enzyme activity level and resulting metabolite fluxes to get optimal responses to different environmental conditions. The user should take into account that any of these expression levels are only crude estimates for the other levels and their effects. However, for most practical purposes it is sufficient if the user knows roughly the order of activity differences between the modeled enzymes, and which important regulatory signals influencing expression levels have to be considered (e.g. an unstable protein or unstable mRNA for a given enzyme should not be neglected). Taking these variables into account, flux distributions can be estimated.

To find the resulting EM activities from the estimated or observed flux distributions, there are, in general, many solutions possible. One could first choose a certain preferred flux mode, adjust its activity, and try to fit the observed flux distribution as accurately as possible. Next, select the next mode and so on. As the modes can be given by different preference schemes, it is absolutely possible that several schemes will fit the distribution equally well.

To find a rational and compact criterion for mode selection, here, we have chosen to first select the modes which are the shortest. It has already been shown in an earlier work [22] that these are the modes which contribute most to gene expression, at least in the central metabolism of *E. coli *(these are actually preferred to be kept by the well connected metabolite choosing routine above). In addition, metabolic webs have been shown to grow selectively around central "hub" metabolites to favor short metabolic paths [18,23].

For calculating EM activities from observed or estimated flux distributions, there are analytical treatments possible based on criteria other than pathway length [20]. However, all experimental measurements have errors. In particular, this applies to gene expression data where detection problems, background and standardization are routine challenges. Similarly, proteome data are selective, and protein levels measured are influenced by factors such as gel resolution, multi-spot detection and similar technicalities. As protein quantifications can only be measured with certain error margins and asinformations about enzyme turnover rates are not always accurate, we do not demand an exact solution to a flux distribution found experimentally (see e.g. Ref [20] for recent advances in this area). We focus on minimizing the difference to the target flux distribution instead. The error function uses the sum of squared differences between calculated enzyme activities (E^C^) and target enzyme activities (E^T^) which is to be minimized and, with R being the number of reactions in the system:



To achieve a fast and robust convergent solution for this error criterion, an evolutionary algorithm (EA) was successfully applied here. The algorithm starts to calculate flux distributions, even if only one enzyme activity or very few are known. A Pareto-optimal solution [24] for such limited experimental data is also found by the evolutionary strategy implemented in YANA.

The algorithm uses a randomly initialized population of 100 individuals with a per feature mutation probability *M*. This depends on the number of features *F *taken into account and the number of iterations *I *already run through, thus introducing a cool-down factor to the mutation probability scaling logarithmically with the number of time steps.



Furthermore, recombination between individuals is achieved by uniform crossover, randomly selecting one of the individuals as a parent for the feature in question. Selection pressure is induced by calculating a rank-based fitness from the square deviation of each individual to the target distribution thus giving each individual a probability *R *to take part in the recombination process that depends upon its rank *r *in the population and the population size *P*.



The evolutionary algorithm routine allows the inclusion of further fitness parameters and helps to fit enzyme activities including these additional constraints. For example, these could be (i) correlations between enzyme expression levels (or just ratios) according to gene-chip experimental results or (ii) constraints based on biochemical data and knowledge on enzyme activities; (iii) metabolite constraints, for instance production of certain amino acids has to be above a certain threshold (given by growth demands or again experimental data), (iv) genetic constraints (certain enzyme genes are known to have modified expression or enzymatic activities), (v) necessary activity or certain levels for specific enzyme pools (e.g. all enzymes connected to redox protection). The fitness function is implemented in such a way, that further positive or negative functions can easily be added with desired weights to the total fitness by the user. Also in that case, the evolutionary algorithm searches for the best possible solution describing the enzyme fluxes with minimum error according to observed enzyme expression data, while including these additional constraints.

In each refinement step, the flux mode fitting routine implemented in YANA selects the shortest modes. If two modes are equal, it picks the better connected inside the network, counting all metabolites according to the reaction they participate in.

For an overview of EA performance, see Results section c).

### Implementation details

For the development of the YANA software package Borland JBuilder 2005 was used in combination with EJ-technologies real-time profiling suite JProfiler. The profiling of the software and the evolutionary algorithm in particular was carried out on a dual Intel Xeon 3.06 GHz CPU with Hyper Threading and 8 GB of RAM. For development and testing, a standard PC with a single 1.7 GHz Pentium 4 processor was used.

The YANA program flow includes the initial editing of a metabolic network in terms of enzymes and their respective substrates and products or, alternatively, loading networks from files in the METATOOL or SBML format.

Subsequently, elementary mode analysis is performed by YANA using the provided precompiled METATOOL binaries. It then parses the resulting output file and presents the arising elementary modes to the user, giving the possibility to retrieve detailed information about a specific elementary mode including partaking enzymes, net reactions, and the stoichiometry involved.

Once the analysis is performed, flux distributions can be visualized in several presentation formats simulating either a virtual spot intensity on a gel, or displaying comparative enzyme copy numbers in a virtual cell. From there on, the user can enter a target flux distribution and YANA tries to adjust the elementary mode activities to approximate the entered flux distribution using the EA.

In any part of the program, diagrams and tables can be printed and exported to graphic files in the portable network graphics (PNG) format or into text files using comma separated values (CSV) for easy import in e.g. Microsoft Excel.

To gain the advantage of platform independence, YANA was implemented using the Java SDK 1.5 and we provide, with our download precompiled METATOOL, binaries for both Windows and UNIX systems. The graphical user interface is based on the SWING Java framework, making strict use of the model-view-controller (MVC) paradigm.

To further add to the usability of the program, support for the Systems Biology Markup Language [25] (SBML Level 2 [26]) was integrated, an XML-based file format which enjoys increasing popularity in current bioinformatics and chemical applications. The software is also able to import and export data in the traditional METATOOL file format.

## Results

### a1) YANA program package

#### Required Inputs

(i) YANA analyses metabolic networks using EMA:

The required input for YANA (and the integrated METATOOL software) to perform an EMA is the set of enzymes and metabolites in the network under study. Their specific reactions and reversibility can be obtained from textbooks and databases. Metabolites must be defined as internal or external according to available biochemical knowledge. Kinetic data, RNA or protein expression data are not required for this part of the analysis.

(ii) For the calculation of flux distributions, the user has to choose as input the activities of the different EMs. If there is no information on this available, YANA assumes all EMs to be equally active. For accurate predictions of enzyme activities experimental data on flux ratios is helpful.

(iii) To analyze how the predicted elementary modes from step 1 fit expression data, some experimental data on protein or gene expression are required. For most accurate predictions in this step, kinetic data on enzyme activities, on translation speed, protein stability and transcription are required. However, YANA needs, as minimum information for predictions, only the estimated activity levels of some of the enzymes involved. Nevertheless, it calculates an optimal solution, mapping the information on enzyme activity available to a predicted EM activity pattern.

#### Program usage and outputs

##### Output

(i) All pathways in the network are calculated, a list of EMs is given, both as enzyme cascades as well as the overall reactions of the elementary modes with educts and products.

(ii) Calculation of specific flux distributions and visualization in form of pie and bar charts and in tabular form.

(iii) A specific EM activity pattern best fitting the user given flux distribution with preferably high activities on short EMs.

##### Usage

The YANA main screen is divided into two parts. On the left hand side, the user is able to enter the metabolites involved in the network with information about whether they are considered internal or external. On the right hand side, the enzymes are defined using the metabolites entered earlier. User actions are usually invoked using the toolbar at the top of the YANA main window. By clicking the analysis button, elementary mode analysis is performed, showing the results in a new frame in a tabular form. YANA gives the user detailed information about the elementary flux modes, their irreversibility constraints, partaking enzymes and so on. The user has the possibility to adjust the activity of each elementary mode on a percent scale using the slider incorporated into the table. By clicking the diagram button, YANA calculates the enzyme activity pattern using the activity vector entered before and displays the results either in a diagram or table format. The flux calculation button brings up a new screen on which the user can enter the target flux distribution for use with the evolutionary algorithm. All table data in YANA can be exported into text files using comma separated values; for convenience the target flux vector can be imported the same way. For further and more detailed information see the readme file that comes with the software package and which is also available via online help.

#### a2) Visualization and analysis of METATOOL output by YANA

**Figure 1:**
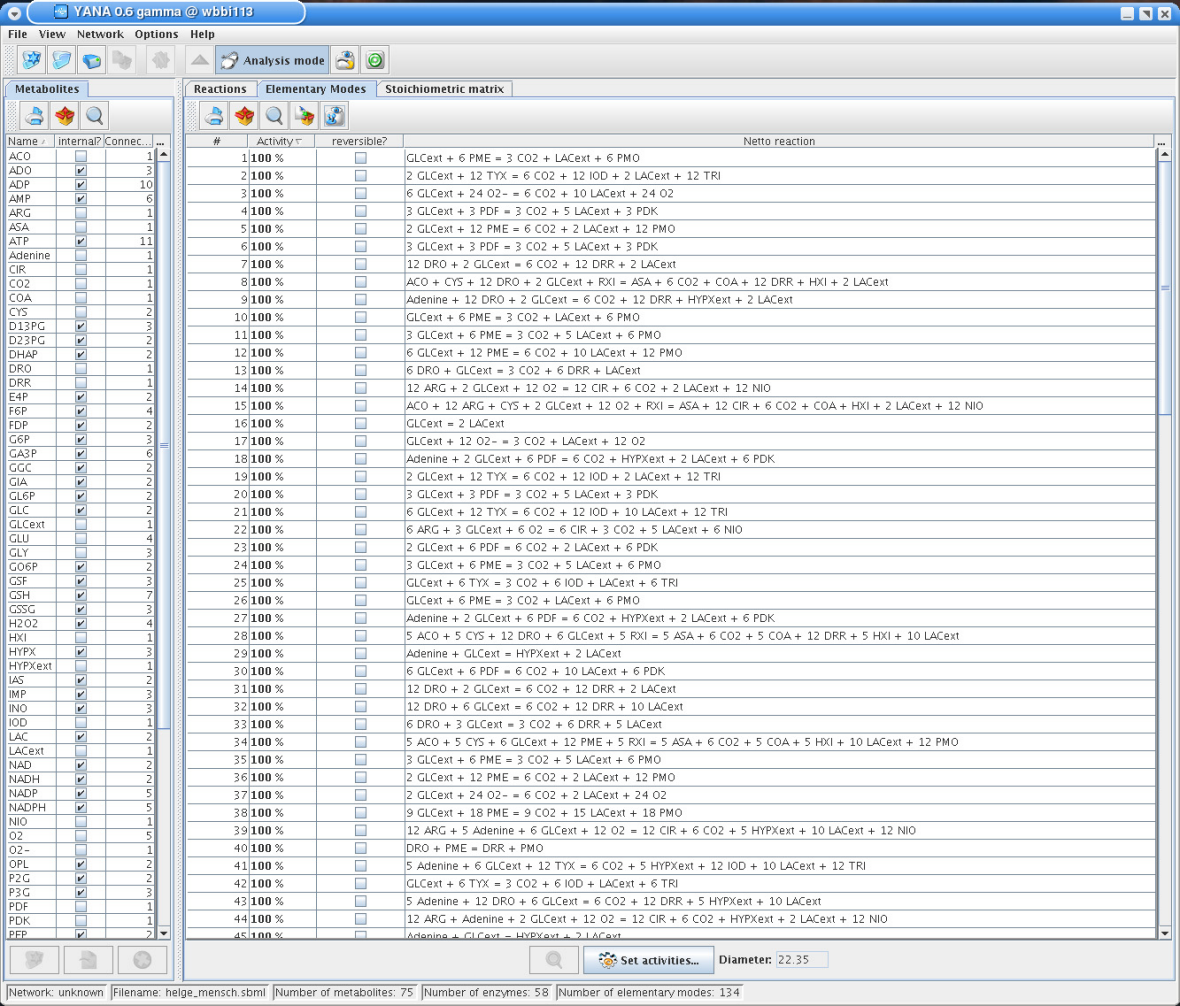
**Screenshot of the GR (glutathione reductase) system in YANA**. The YANA main screen showing the GR redox network involving 75 metabolites (left side view) and 58 enzymes (right side view), resulting in 134 flux modes (not shown here).

To demonstrate the YANA package we use a running example (Figure 1) of the human redox metabolism (75 metabolites (46 internal, 29 external) and 58 enzymes), around the central enzyme glutathione reductase [see Additional file 1]; see also Ref [14]; extended from Ref [27]).

Under stationary conditions, this system yields a total of 134 EMs. From these, 46 include glutathione reductase, 117 involve energy consumption (ATP), whereas 128 involve redox reactions. The complete listing of these modes is given in supplementary material [see Additional file 1].

#### b1) YANA dissects and analyzes a metabolic web according to well connected metabolites

To illustrate the complexity reduction power of the YANA strategies we performed a series of EMAs with rising threshold values using both threshold types.

##### Dissection (Table 1)

Using a threshold of 7 (metabolites participating in more than seven reactions are considered external), only membrane phosphorylation is placed in a separate sub-network. A threshold of 5 splits the system into seven sub-networks with instructive specific biochemical functions and flux modes: (i) a well connected sub-network includes salvage pathway, pentose phosphate cycle, NOS, SOD and redox protection by uric acid and GSH; other sub-networks are (ii) GSH synthesis, (iii) GSH degradation and GSH protection of protein groups, (iv) membrane phosphorylation (as with threshold 7), (vi) lower glycolysis (trioses), (vii) adenylate kinase. Threshold 3 splits these sub-networks further into a total of 18 components, e.g. the well connected sub-network (i) is now put into its single pathways as named above.

##### Hub metabolites (Table 2)

The complex system of 134 elementary modes is first reduced to a 87-mode system (GR modes and pathways which are more central than other redox enzyme paths remain, if the threshold is set to 5 reactions). Only 24 modes remain if the threshold is set to the best connected metabolite, the currency metabolite ATP. The very short diameter obtained shows that this analysis zooms in on hub metabolites [18] and well connected next-neighbor reactions, showing the quickly equilibrated central parts of the system which one could consider more (high threshold) or less (low threshold) as external and well buffered central pools, the most pronounced being the reactions with the central currency metabolite ATP.

#### b2) YANA translates EM activities into specific flux distributions

Table 4 shows the calculated flux distribution for the system under study if all EMs are considered equally active (100% activity).

In the example, GR as a central enzyme of the network has an activity of 399. Besides this, the most active enzymes are: GAPDH (598), PGM (598), LDH (598), PGK (560), PK (598) and EN (598), as a parts of glycolysis, and the enzymes G6PD (576); PGLase (576) and GL6PDH (576), as components of the oxidative part of the pentose phosphate pathway. For the obtained flux distribution, we notice a tight connection between glycolysis and the glutathione reductase metabolism. The main pathways of glycolysis and PPP supply energy and reduction equivalents for strong redox protection provided by the glutathione reductase network. In contrast, several other enzymes are downregulated, in particular, those which use uric acid as an antioxidant as well as catalase.

**Figure 2:**
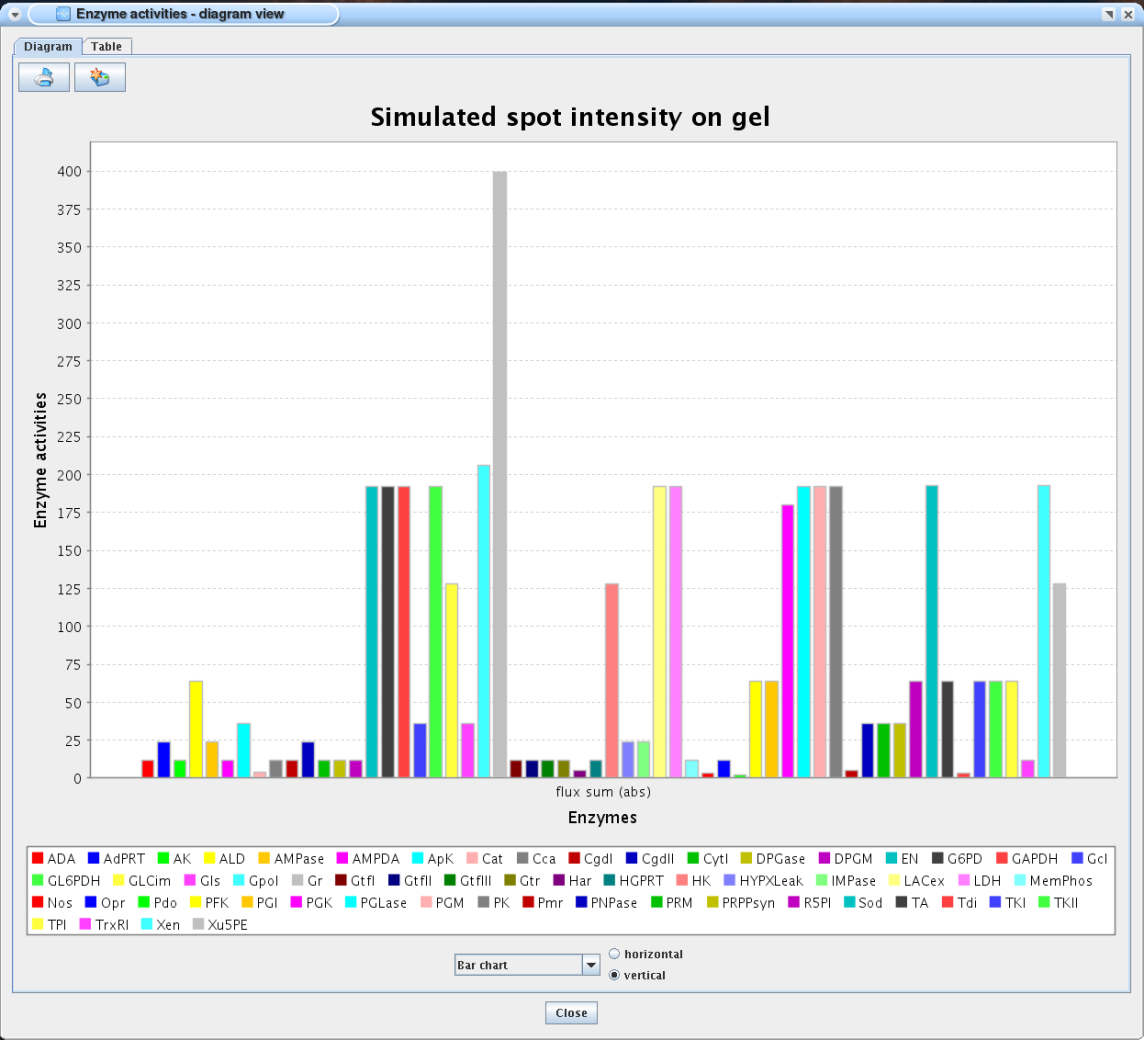
**Screenshot of the simulated enzyme activities diagram**. Diagram of simulated spot intensities on a gel, after activation of GR containing elementary modes. Obviously glutathione reductase is indeed most active whereas other enzymes not involved in the core GR part of the system are downregulated.

The program also quickly calculates and visualizes flux distributions for any other chosen EM activities as given in Table 4. Thus, one notices that selective activation of EMs related to the pentose phosphate pathway leads to similar results as above. Setting only HGPRT-containing modes at a maximum activity (and all others to 0%) gives a more selective response with several enzymes completely deactivated. Finally, when all modes containing glutathione reductase are active, the graph shows the central position of GR in the network by a peak, and underlines even more the importance of critical energy providing pathways for redox protection (Figure 2).

#### c) Out of (partially) known flux distributions, YANA predicts and identifies EM activities with minimal error

Using our illustration example, we give

a) the results for the situation where only the enzyme fluxes for glycolytic enzymes are all set to 100 (equal activity, for convenience assumed to represent international enzyme units [micromol/minute]) and all others are known to be at zero.

b) The same as before, but all other fluxes are unknown or simply have not been measured (the enzyme activity is then set to -1 in order to indicate lack of knowledge).

Situation a) reveals a flux distribution in which, after upregulation of glycolytic enzymes, the three enzymes forming the oxidative part of PPP are also highly active. In addition, glutathione reductase (GR), NO synthase (NOS) and TrxRI (thioredoxin reductase) are upregulated as well, showing that a major part of the metabolite flux uses the path from glycolysis via oxidative PPP to redox protection enzymes. Not connected to glycolysis at all, and thus set to zero activity, are again the use of uric acid as an antioxidant and catalase.

Situation b) – a scenario where the measured data are similar but more incomplete – gives similar results, underlining that glycolysis or its side-products are important for many reactions in this network. For this case, uric acid as anti-oxidant and catalase are predicted not to be used. Detailed results for both situations are given in supplementary material. [see Additional file 1].

For comparison, experimental data on the activity of glutathione reductase and the connected redox network have been reported by Krauth-Siegel et al. (1996) [28] and others. The concentration of glutathione reductase is approx. 0.2 μM in human red blood cells and in the cytosol of various eukaryotic cells [28,29]. In erythrocytes, this corresponds to a maximal enzyme activity of 2 U/ml at 25°C. Assuming that the concentration of the substrate glutathione disulfide is 1 to 10 μM under physiological conditions, the turnover of substrate can be estimated to be 30 μM/min to 270 μM/min (30 mU/ml to 270 mU/ml).

Transcriptome analyses have been reported for antioxidant proteins of the malaria parasite *Plasmodium falciparum *in its various developmental stages [30]. The other side of the coin, the proteomics of oxidatively modified proteins has been reviewed by Ghezzi and Bonetto (2003) [31].

The still sparse and incomplete data support the scenarios discussed here, in particular regarding the high activity of glutathione reductase modes as well as the importance of energy providing reactions. However, a detailed kinetic and experimental metabolic flux analysis of the whole system has not yet been achieved.

The convergence criterion for the EA was to achieve a sum-of-squares error of less then 5% of the best evolved flux distribution to the target flux distribution. Regarding measurement or experimental errors and constraints, the user is alerted in case measurements are incompatible with the calculated theoretical flux distribution but also about which data are responsible for maximizing the difference between observed and calculated flux distribution.

In Table 3 EA convergence is tested using randomly generated flux distributions as test datasets, working on our example system with 134, 48 and 24 modes.

Using the example network above, with a threshold of 8, more than 50% convergence could be reached after 100 iterations (22 seconds).

## Discussion

After its conceptual description [2], Elementary Mode Analysis has continuously been improved including new algorithms [12,19,32], visualization (php-Metatool [33]) and dissection algorithms [13,14]. Computation of elementary modes and visualization of these is also feasible by the program FluxAnalyzer [17]. Furthermore, alternative approaches also allow enumerating feasible routes in complex metabolic networks, for instance extreme pathway analysis [4] and hierarchical decomposition [34]. All these further implementations and algorithmic developments have specific advantages, but also limitations.

The current software package allows user-friendly post-processing of the METATOOL output. In particular visualization of the modes, editing metabolites and reactions, and graphical comparisons of enzymes and their involvement in reactions of the metabolic network are available for the user. YANA is a stand-alone visualization tool with its focus on user intervention, the quick comparison of results and thorough data exchange capabilities. In contrast, there are a number of more complex and integrated packages available such as GEPASI [15,35] which have less visualization options and offer other calculation possibilities.

For addressing the major problem of combinatorial explosion of the number of EMs in complex networks, YANA implements a decomposition method proposed earlier [13]. In this method, all highly connected metabolites are set to external status. Moreover, a new simplification strategy is offered to reduce complex metabolic networks. Earlier studies on metabolite databases show that the well connected "hub" metabolites dominate the overall architecture of a metabolic web and represent its core [18]. Here we offer the option to consider only those reactions where well connected metabolites are involved – the threshold can be chosen by the user. In fact, the results here show that such a procedure reduces a metabolic web considerably. This is particularly useful to dissect and put apart those larger parts of the metabolic web which are not well connected, so that they do not add to the central part of this metabolic map.

Metabolic fluxes are difficult to measure. YANA offers a specific approach to correlate metabolic fluxes with EM activities. Alternative algorithms for such an effort have been proposed [20,22]. The YANA routine offers several advantages. Firstly, most experimental data on protein or gene expression are always prone to errors and noisy. To account for this, in YANA no exact EM activity solution for the corresponding flux distribution is sought. Instead, the experimental input is critically examined in regard to whether it is realistic and can be satisfied by any combination of EMs. Next, the error between the observed values of enzyme fluxes and the theoretical calculated flux distribution is minimized. Accordingly, YANA also accepts rather incomplete measurements, for instance, when only two enzyme flux values are known. Furthermore, the evolutionary strategy allows incorporating any further user-desired multiple constraints into the fitness function.

The calculated EM activity pattern should additionally satisfy metabolite restrictions, as well as growth or genetic considerations on the enzyme or metabolite profile. Further constraints, which might be considered, are, for example, expression constraints dependent on promoter structure, RNA stability or protein stability. In spite of this flexibility, the evolutionary strategy converges swiftly to a solution. The great advantage of this is that we have both robust optimization and already take into account that there is noise, and that no perfect solution is possible. If desired, more criteria could be added with ease to the EA.

## Conclusion

YANA adds a compact, user-friendly software package to the analysis of metabolic webs, offering several new implementations for typical challenges in such analyses including modeling of expression data. The results illustrate the application for a central redox network around glutathione reductase. Further developments will consider additional regulatory constraints profiting from the evolutionary strategy applied as well as a graphical editor for the metabolic networks including dedicated algorithms for the automatic layout of the graphs.

## Availability and Requirements

All software and documentation are available for download at .

The package requires at least Java Runtime Environment (JRE) Version 1.5.0 and the following libraries, which are included in the download bundle and can be found in the /lib subdirectory:

• GenJava-CSV (© 2003, Henri Yandell)

• Jakarta Common Collections 3.1 (© 2004, The Apache Software Foundation)

• JFreeChart 0.9.21 (© 2004, Object Refinery Limited and Contributors)

• JigCell Modelbuilder (© 2004, Virginia Polytechnic Institute and State University)

• JMat 5.0 (© 2004, Yann Richet)

• Mosfet Liquid L&F (© 2004, Miroslav Lazarevic)

• Noia KDE 1.00 (© Carles Carbonell Bernado)

All libraries are licensed under either GNU General Public License (GPL) [36], Lesser GNU General Public License (LGPL) [37], BSD OpenSource License [38], DARPA BioComp OpenSource License, or other proprietary open source licenses that allow the use, redistribution, and modification of the application or parts of it. The copyright stays with the corresponding authors.

A 1.4 GHz CPU and 256 MB RAM are recommended for running the YANA software package. Installation requires at least 30 MB of hard disk space. YANA is supposed to run on any 32-bit Windows or Linux platform.

## List of abbreviations

• EMA – Elementary Mode Analysis

• EM – Elementary Mode, also known as Elementary Flux Mode or Flux Mode

• EA – Evolutionary Algorithm

## Authors' contributions

All authors read and approved the ms and made critical comments, adding to the final version presented here. In addition they contributed

RS: Architecture and implementation, graphical design, design of user interface.

PM: Tested and wrote an early implementation of the software and simplification routine.

AVK: Tested YANA, Metatool expertise, compatibility with Metatool.

BE: Provided theoretical insights and chemistry knowledge.

RHS: Provided experimental insights and discussion points.

SS: Biophysical knowledge, expertise in flux balance analysis, metabolic modelling and interpretation of obtained modes as well as for algorithm strategy.

TD: Concept; plan for the software and strategy, lead and guided the study.

## Supplementary Material

Additional File 1 Metabolic network around GR reductase and flux distribution examples (Microsoft Excel 2003): The file contains the complete metabolic network used for elementary mode analysis including the metabolites, reactions / enzymes and elementary modes. Additionally, two flux distributions for upregulated glycolysis are given in the file, as discussed in the main section of the article. Click here for file

## Figures and Tables

**Table 1:** Simplification of the GR system by dissection at highly connected metabolites (cutting)

**Threshold**	**No. of elem. modes**	**GR involved**	**ATP involved**	**Redox reactions**	**Diameter**
>11	134	46 (34%)	117 (87%)	128 (95%)	22.35
7	215	68 (31%)	131 (60%)	199 (92%)	22.26
5	35	4 (11%)	18 (52%)	16 (47%)	6.17
3	10	0 (0%)	5 (50%)	2 (20%)	3.0

**Table 2:** Simplification of the GR system by concentration on highly connected metabolites (centralization)

**Threshold**	**No. of elem. modes**	**GR involved**	**ATP involved**	**Redox reactions**	**Diameter**
0	134	46 (34%)	117 (87%)	128 (95%)	22.35
5	87	22 (25%)	45 (52%)	32 (37%)	2.75
10	24	0 (0%)	24 (100%)	0 (0%)	2.38

**Table 3:** EA performance for three levels of complexity

**No. of elementary modes**	**Average time to convergence**
134	1147.3 sec
48	81.7 sec
24	13.2 sec

**Table 4:** Individual enzyme activities summed over all elementary modes Calculation of individual enzyme activities according to a given flux distribution: The 134 modes obtained from the input system [see Additional file 1] are all assumed to be active with standard (1 flux unit) activity. Alternatively, fractions of full activity of individual modes (given in percentages) can be set by the user and included in the calculation. For the standard flux vector, the total enzyme activities are calculated by YANA as follows (arbitrary units, only relative fluxes are calculated):

**Name**	**Act. (a.u.)**	**Name**	**Act. (a.u.)**	**Name**	**Act. (a.u.)**	**Name**	**Act. (a.u.)**
ALD	203.0	ApK	111.0	DPGM	38.0	EN	598.0
GAPDH	598.0	GpoI	209.0	Gr	399.0	HYPXLeak	74.0
LACex	598.0	PGI	203.0	PGK	560.0	PGLase	576.0
PGM	598.0	Pmr	201.0	PNPase	111.0	PRM	111.0
PRPPsyn	111.0	R5PI	192.0	TA	192.0	TKI	192.0
TKII	192.0	TPI	203.0	TrxRI	589.0	Xu5PE	384.0
ADA	37.0	AdPRT	74.0	AK	38.0	AMPase	75.0
AMPDA	37.0	Cat	6.0	Cca	37.0	CgdI	37.0
CgdII	75.0	CytI	38.0	DPGase	38.0	G6PD	576.0
Gcl	112.0	GL6PDH	576.0	GLCim	395.0	Gls	112.0
GtfI	37.0	GtfII	37.0	GtfIII	38.0	Gtr	37.0
Har	7.0	HGPRT	37.0	HK	395.0	IMPase	74.0
LDH	598.0	MemPhos	38.0	Nos	196.0	Opr	38.0
Pdo	99.0	PFK	203.0	PK	598.0	Sod	196.0
Tdi	196.0	Xen	196.0				
